# Ultrafine Vacancy-Rich Nb_2_O_5_ Semiconductors Confined in Carbon Nanosheets Boost Dielectric Polarization for High-Attenuation Microwave Absorption

**DOI:** 10.1007/s40820-023-01151-0

**Published:** 2023-07-14

**Authors:** Zhe Su, Shan Yi, Wanyu Zhang, Xiaxi Xu, Yayun Zhang, Shenghu Zhou, Bo Niu, Donghui Long

**Affiliations:** 1 Shanghai Key Laboratory of Multiphase Materials Chemical Engineering, School of Chemical Engineering, East China University of Science and Technology, Shanghai, 200237 People’s Republic of China; 2 Key Laboratory for Specially Functional Materials and Related Technology of the Ministry of Education, East China University of Science and Technology, Shanghai, 200237 People’s Republic of China

**Keywords:** Electromagnetic wave absorption, Nb_2_O_5_ semiconductor, Dielectric polarization loss, Oxygen vacancy, Nb_2_O_5_– carbon hetero-interface

## Abstract

The integration of nano-semiconductors into electromagnetic wave absorption materials is a highly desirable strategy for intensifying dielectric polarization loss; achieving high-attenuation microwave absorption and realizing in-depth comprehension of dielectric loss mechanisms remain challenges. Herein, ultrafine oxygen vacancy-rich Nb_2_O_5_ semiconductors are confined in carbon nanosheets (ov-Nb_2_O_5_/CNS) to boost dielectric polarization and achieve high attenuation. The polarization relaxation, electromagnetic response, and impedance matching of the ov-Nb_2_O_5_/CNS are significantly facilitated by the Nb_2_O_5_ semiconductors with rich oxygen vacancies, which consequently realizes an extremely high attenuation performance of − 80.8 dB (> 99.999999% wave absorption) at 2.76 mm. As a dielectric polarization center, abundant Nb_2_O_5_–carbon heterointerfaces can intensify interfacial polarization loss to strengthen dielectric polarization, and the presence of oxygen vacancies endows Nb_2_O_5_ semiconductors with abundant charge separation sites to reinforce electric dipole polarization. Moreover, the three-dimensional reconstruction of the absorber using microcomputer tomography technology provides insight into the intensification of the unique lamellar morphology regarding multiple reflection and scattering dissipation characteristics. Additionally, ov-Nb_2_O_5_/CNS demonstrates excellent application potential by curing into a microwave-absorbing, machinable, and heat-dissipating plate. This work provides insight into the dielectric polarization loss mechanisms of nano-semiconductor/carbon composites and inspires the design of high-performance microwave absorption materials.

**Supplementary Information:**

The online version contains supplementary material available at 10.1007/s40820-023-01151-0.

## Highlights

- Ultrafine oxygen-vacancy-rich Nb_2_O_5_ semiconductors have been incorporated into carbon nanosheets for high-attenuation microwave absorption (−80.8 dB, > 99.999999% wave absorption).
- Nb_2_O_5_ semiconductors with abundant oxygen vacancies significantly facilitate polarization relaxation, electromagnetic response, dipole polarization, and interfacial polarization.
- Multiple reflections and scattering dissipation are enhanced by the unique 2D lamellar morphology.

**Supplementary Information:**

The online version contains supplementary material available at 10.1007/s40820-023-01151-0.

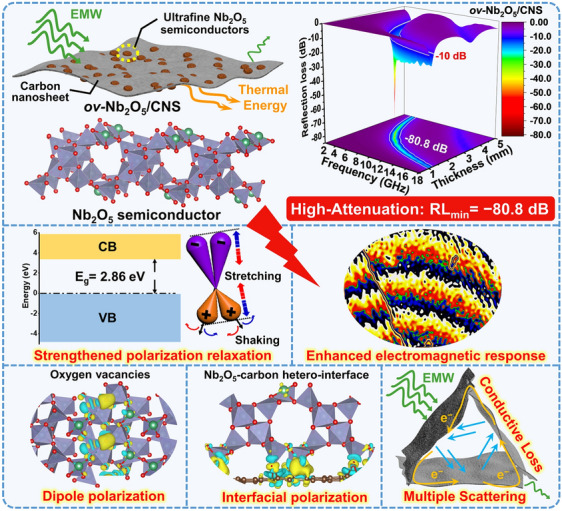

## Introduction

With the flourishing development of wireless communication, particularly fifth-generation (5G) communication technology, serious concerns about electromagnetic radiation, electromagnetic interference, and electromagnetic pollution have arisen due to rampant electromagnetic wave (EMW) signals [1–3]. Microwave absorption materials (MAMs) with exceptional abilities to absorb and dissipate electromagnetic energy have non-negligible potential for addressing electromagnetic hazards [4–6]. The EMW attenuation performance characteristics of MAMs are closely associated with the dielectric/magnetic loss parameters (complex permittivity and permeability) and morphologies. Hence, constructing MAMs with elaborately optimized dielectric/magnetic loss properties and absorption-promoted morphology has been considered an effective EMW absorber design criterion [7–9].

To obtain MAMs with excellent dielectric/magnetic loss characteristics, researchers have made great efforts to construct multicomponent nanocomposites [4, 10–12]. This effort is because the EMW absorption properties of nanocomposites can be significantly optimized by integrating additional dielectric/magnetic components, thereby introducing many new attenuation mechanisms. For instance, Gao et al. [13] reported that Cu-intercalated MoS_2_ with carbon modification can achieve synergistic multiple polarization. Su et al. [14] demonstrated a ternary-alloy FeCo_2_Ni/carbon composite, by which EMW absorption can be enhanced through the integration of magnetic–dielectric complementary attenuation capabilities. Li et al. [15] fabricated NiO/Ni particles on N-doped hollow carbon spheres with improved conduction loss and polarization loss. Xie et al. [16] synthesized oxygen vacancy-containing MoO_3_/PPy composites for electromagnetic wave absorption, showing enhanced electrical conductivity and dielectric loss. Conversely, the structure and geometry significantly influence both the interfacial polarization losses of MAMs and the penetration and scattering paths of EMWs, playing essential roles in the attenuation of EMW energy [17, 18]. Therefore, to enhance interfacial polarization loss and prolong the energy attenuation path of EMW, researchers have made great efforts to design and construct absorbing materials with versatile structures in special dimensions [19–21]. For example, nanotubes in one-dimensional (1D) structures [22–24], nanosheets in two-dimensional (2D) structures [8, 18, 25], and three-dimensional (3D) hollow/yolk-shell structures have been reported [26, 27]. Generally, constructing multicomponent nanocomposites and designing various nano/microstructures are the main aspects of MAM preparation.

As a typical class of carbonaceous MAMs, carbon nanosheets (CNSs) possess extensive 2D lamellar interfaces that facilitate interfacial polarizations and multiple scatterings and are suitable for active composition regulations [28–30]. Integrating dielectric-type components into CNSs always holds broad research interest [31, 32]. The dielectric components with complex loss mechanisms of dielectric resonance, multiple polarization relaxation and abundant dipole-induced polarization can positively boost EMW attenuation [33–36]. As a prominent dielectric material, semiconductors (e.g., TiO_2_ [37], SiC [38], and Nb_2_O_5_ [38]) exhibit distinctive electronic properties, such as electron hopping and electron transport, which can be effectively modulated through particle size regulation, doping design, and defect engineering to tune the polarization loss characteristics [16, 27, 39]. Commonly, nano-semiconductors embedded in carbonaceous substrates tend to form abundant nano-heterointerfaces. The original electric balance is disrupted at these nano-interfaces due to the different electronic properties of interfacial atoms at heterointerfaces. As a result, an increased number of polarization loss sites are induced by the consequent local charge separation and dipole formation under the alternative field. Given the respective benefits of dielectric semiconductors and CNSs, nano-semiconductor-anchored CNS nanocomposites can be novel EMW absorbers. In this context, controllable synthesis and insight into the dielectric loss mechanisms of nano-semiconductor-anchored CNS nanocomposites are urgently required to meet the demands of real-world applications.

Herein, we present ultrafine (~ 10 nm) oxygen vacancy-rich Nb_2_O_5_ nano-semiconductors confined in carbon nanosheets (*ov-*Nb_2_O_5_/CNS) as high-attenuation EMW absorption materials. The innovative synthesis route of direct carbonization of an organic Nb^5+^-gluconate precursor with spontaneous foaming characteristics promises the efficient constitution of a 2D morphology and produces abundant Nb resources to form Nb-based nanoparticles (Nb_2_O_5_ or NbC). Compared with NbC conductors, semiconductive Nb_2_O_5_ facilitates polarization relaxation and the electromagnetic response and triggers stronger local charge separation at the Nb_2_O_5_–carbon hetero-interface. Interestingly, the presence of oxygen vacancies endows Nb_2_O_5_ nanocrystals with extensive charge separation sites to reinforce electric dipole polarization inside the semiconductor. Moreover, due to the 2D lamellar conductive carbon skeleton, satisfactory conductive loss, abundant macro-interfacial polarization and multiple scattering are realized to attenuate EMW energy. Consequently, *ov-*Nb_2_O_5_/CNS with boosted dielectric polarization losses exhibits superior EMW absorption with an ultralow minimum reflection loss (*RL*_min_) of − 80.8 dB (> 99.999999% wave absorption) at 7.11 GHz (2.76 mm) and a wide effective absorption bandwidth of 3.37 GHz at 1.30 mm. In addition, the composite possesses excellent application potential by curing with cyanate resin to form a microwave-absorbing *ov-*Nb_2_O_5_/CNS-cyanate plate. These results verify that *ov-*Nb_2_O_5_/CNS is expected to be an excellent microwave absorber when practically applied.

## Experimental Section

### Materials

Ammonium niobate (V) oxalate hydrate, ammonium hydroxide solution, and anhydrous ethanol were purchased from Aladdin Co. Ltd. Gluconic acid solution was purchased from Energy Chemical. All reagents were used as received without further purification. Deionized water was used throughout the experiments.

### Preparation of Nb^5+^-Gluconate Precursor

Nb^5+^-gluconate was obtained from a neutralization reaction of gluconic acid and Nb(OH)_5_. Briefly, 24.71 g (≈0.08 mol) ammonium niobate (V) oxalate hydrate was added to 100 mL deionized water at 80 °C and continuously stirred until it was completely dissolved. Then, ammonium hydroxide solution (25 wt%) was added to the above solution to adjust the pH level to pH > 7. The resulting white Nb(OH)_5_ precipitate was centrifuged and washed three times with deionized water. A total of 160 g gluconic acid solution (50 wt%) and 20 mL deionized water were added to the above fresh Nb(OH)_5_ paste. After continuous stirring at 80 °C, Nb(OH)_5_ was completely dissolved, and a transparent Nb^5+^-gluconate solution was obtained. Finally, the obtained Nb^5+^-gluconate solution was added dropwise into anhydrous ethanol to crystallize. Nb^5+^-gluconate was separated from the ethanol solution by filtration, washed 3 times with anhydrous ethanol, and dried overnight.

### Preparation of ***a-***Nb_2_O_5_/CNS, ***ov-***Nb_2_O_5_/CNS, ***c-***NbC/CNS, and ***wc-***NbC/CNS Composites

The Nb^5+^-gluconate precursor was simply carbonized at different temperatures (700, 800, 1000, and 1200 °C) for 2 h under a N_2_ atmosphere with a heating rate of 3 °C min^−1^ to obtain *a-*Nb_2_O_5_/CNS, *ov-*Nb_2_O_5_/CNS, *c-*NbC/CNS, and *wc-*NbC/CNS composites. *a-*Nb_2_O_5_/CNS, *ov-*Nb_2_O_5_/CNS, *c-*NbC/CNS, and *wc-*NbC/CNS denote amorphous Nb_2_O_5_/CNS, oxygen vacancy Nb_2_O_5_/CNS, crystallized NbC/CNS, and well-crystallized NbC/CNS, respectively.

### Material Characterizations

The morphologies of the samples were observed by field emission scanning electron microscopy (FESEM; Nova NanoSEM 450) and field emission transmission electron microscopy (FETEM; JEM-2100F). Off-axis electron holography was observed by Lorentz-transmission electron microscopy (JEM-2100F). Thermogravimetric analysis (TGA; PerkinElmer TGA 4000) was conducted at an N_2_ or airflow of 50 mL min^−1^, with a heating rate of 10 °C min^−1^. The X-ray diffraction (XRD) patterns were obtained on a Rigaku D/max 2550 diffractometer operating at 40 kV and 20 mA using Cu Kα radiation (λ = 1.5406 Å). X-ray photoelectron spectrometry (XPS) (Thermo Scientific ESCALAB Xi +) was used to determine the surface elemental compositions of the samples. The Raman spectrum was tested by Invia Reflex with a wavelength of 532 nm. Nitrogen adsorption/desorption isotherms were measured at 77 K with a Quadrasorb SI analyzer. Electron paramagnetic resonance (EPR) spectra were measured on a Bruker EMXPLUS spectrometer at room temperature. Microcomputer tomography (micro-CT; Xradia 520 Versa) was used to acquire sliced images.

### Electromagnetic Measurement

The samples were uniformly dispersed in a paraffin matrix at 40 wt%, which was fabricated into a cylindrical sample with an inner diameter of 3.04 mm, an outer diameter of 7.00 mm, and a thickness of 2.00 mm. A vector network analyzer (VNA; Agilent E5071C) was used to measure the complex permittivity and permeability characteristics in the range of 2–18 GHz with the coaxial line method.

### Preparation of ***ov-***Nb_2_O_5_/CNS-cyanate Plate

First, 2.04 g *ov-*Nb_2_O_5_/CNS was mixed with 10.20 g cyanate ester (*ov-*Nb_2_O_5_/CNS: cyanate ester = 1:5). Then, 0.05 g curing agent of dibutyltin dilaurate was added to the above mixture and continuously stirred to form a uniform mixture resin. The *ov-*Nb_2_O_5_/CNS-cyanate resin was curved in an 80 mm × 40 mm mold at 80 °C to form the *ov-*Nb_2_O_5_/CNS-cyanate plate. The plate was further machined to the desired thickness and size.

### Density Functional Theory Calculations

First-principles calculations were employed to perform all density functional theory (DFT) calculations within the generalized gradient approximation (GGA) using the Perdew–Burke–Ernzerhof (PBE) formulation. The projected augmented wave (PAW) potentials were chosen to describe the ionic cores and consider valence electrons using a plane wave basis set with a kinetic energy cutoff of 500 eV (for the energy band structure) or 520 eV (for the charge density distribution). Partial occupancies of the Kohn−Sham orbitals were allowed by using the Gaussian smearing method with a width of 0.05 eV. The electronic energy was considered self-consistent when the energy change was smaller than 10^−4^ eV (for the energy band structure) or 10^−5^ eV (for the charge density distribution). A geometry optimization was considered convergent when the energy change was smaller than 0.04 Å^−1^ (for the energy band structure) or 0.03 eV Å^−1^ (for the charge density distribution). For the energy band structure, Grimme’s DFT-D3 methodology was used to describe the dispersion interactions. For charge density distributions, in our structure, U correction was used for Nb (3.91 eV) atoms. The vacuum spacing in a direction perpendicular to the plane of the structure was 20 Å for the surfaces. Brillouin zone integration was performed using 3 × 3 × 2 (for the energy band structure) or 3 × 3 × 1 (for the charge density distribution) Monkhorst–Pack *k*-point sampling for a structure.

## Results and Discussion

### Preparation and Structure Characterizations

**Fig. 1:**
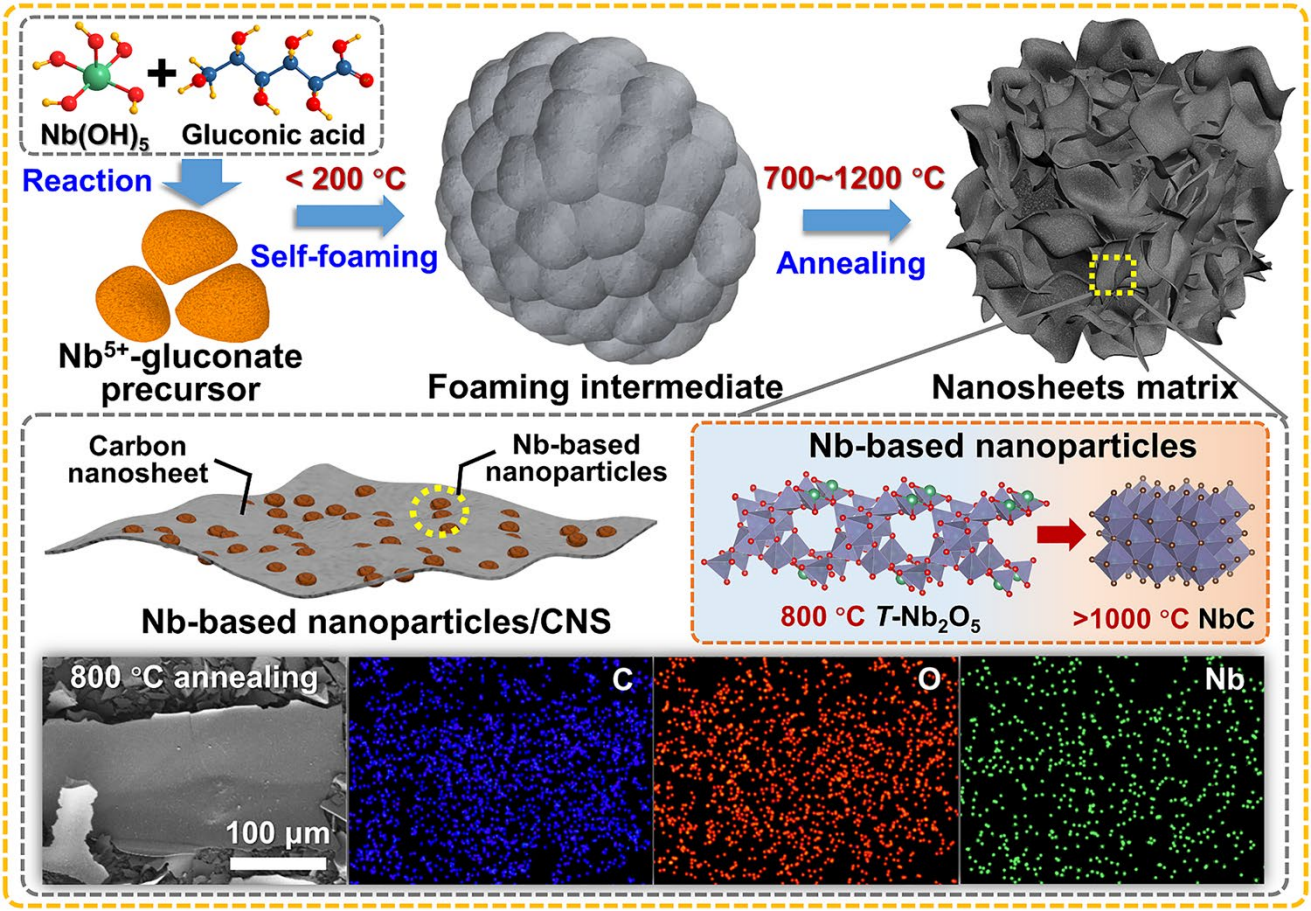
Schematic illustration of the synthesis of two-dimensional Nb_2_O_5_ and NbC nanoparticle/carbon nanosheet composites

The synthesis of Nb_2_O_5_ and NbC nanoparticle/carbon nanosheet composites is achieved for the first time via direct carbonization of the organic Nb^5+^ gluconate (Nb-GlcA) precursor, as illustrated in Fig. 1. The Nb-GlcA precursor is tactfully prepared through the highly efficient neutralization reaction between gluconic acid and freshly precipitated Nb(OH)_5_, as expounded upon in the Experiment section. Notably, the Nb-GlcA precursor exhibits a unique self-foaming characteristic, leading to a very high volume expansion upon carbonization, as shown in Fig. S1. During the initial thermal treatment stage (< 200 °C), the precursor undergoes a morphological transformation from compact bulks to intermediates with closed-cell structures (Fig. S2). The closed-cell foaming structures ultimately develop into 2D lamellar structures for the final product. With further high-temperature annealing (700–1200 °C), organic gluconic carbohydrates and Nb^5+^ ions undergo structural rearrangement and transform into carbonaceous nanocomposites embedded with different types of Nb-based nanoparticles. The contents of Nb-based nanoparticles in these samples are ≈34–40 wt%, according to the thermogravimetric analysis results (Fig. S3). From scanning electron microscopy (SEM) observations, all the composites feature a fragmented lamellar morphology with a large planar size (> 50 mm) (Fig. S4). Moreover, C, O, and Nb are homogeneously distributed in the 2D hybrid skeleton, according to X-ray energy-dispersive spectroscopy (EDS) (Fig. S5).

**Fig. 2:**
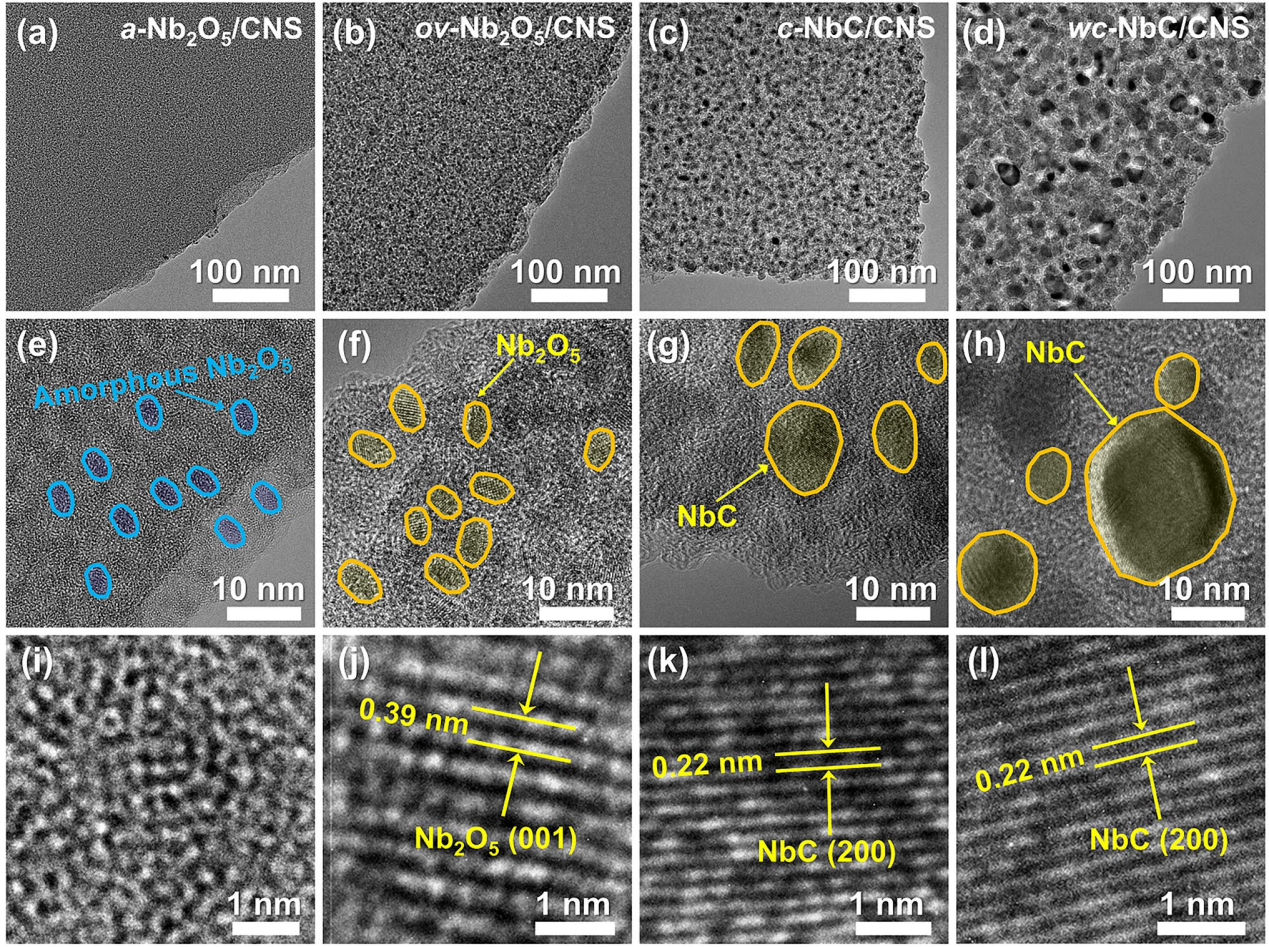
**a–d** TEM images, **e–h** high-resolution TEM images, and **i–l** lattice fringe images of *a-*Nb_2_O_5_/CNS, *ov-*Nb_2_O_5_/CNS, *c-*NbC/CNS, and *wc-*NbC/CNS composites

The annealing temperature determines the microstructure and crystallography characteristics of the as-obtained composites, which are investigated through transmission electron microscopy (TEM) (Fig. 2). For all composites, plenty of Nb-based nanoparticles are homogeneously distributed in the 2D lamellar amorphous carbon skeletons (Fig. 2a–d). As the annealing temperature increases from 700 to 1200 °C, the size of Nb-based nanoparticles grows from less than 5 nm to approximately 20 nm (Fig. 2e–h). Simultaneously, the Nb-based nanoparticles undergo phase transitions from amorphous Nb_2_O_5_ (700 °C) to crystallized Nb_2_O_5_ (800 °C) and are eventually reduced to NbC (> 1000 °C), as evidenced by the high-resolution TEM (HRTEM) images in Fig. 2i–l. Specifically, the amorphous nature of Nb_2_O_5_ nanoparticles in *a-*Nb_2_O_5_/CNS (700 °C) is evidenced by the absence of distinct crystal lattice fringes, as depicted in Fig. 2i. Upon increasing the annealing temperature to 800 °C, the Nb_2_O_5_ nanoparticles in *ov-*Nb_2_O_5_/CNS are crystallized from the carbon skeleton, which demonstrates a lattice spacing of 0.39 nm corresponding to the (001) plane of the orthorhombic Nb_2_O_5_ crystal (Fig. 2j). The reduction in Nb_2_O_5_ nanoparticles by the carbon skeleton is facilitated by their ultrafine size, enabling the process to occur at a relatively low temperature of 1000 °C. Hence, the crystallized nanoparticles in *c-*NbC/CNS (1000 °C) and *wc-*NbC/CNS (1200 °C) with an average lattice spacing distance of 0.22 nm are indexed to the crystalline cubic NbC (200) plane (Fig. 2k–l).

**Fig. 3:**
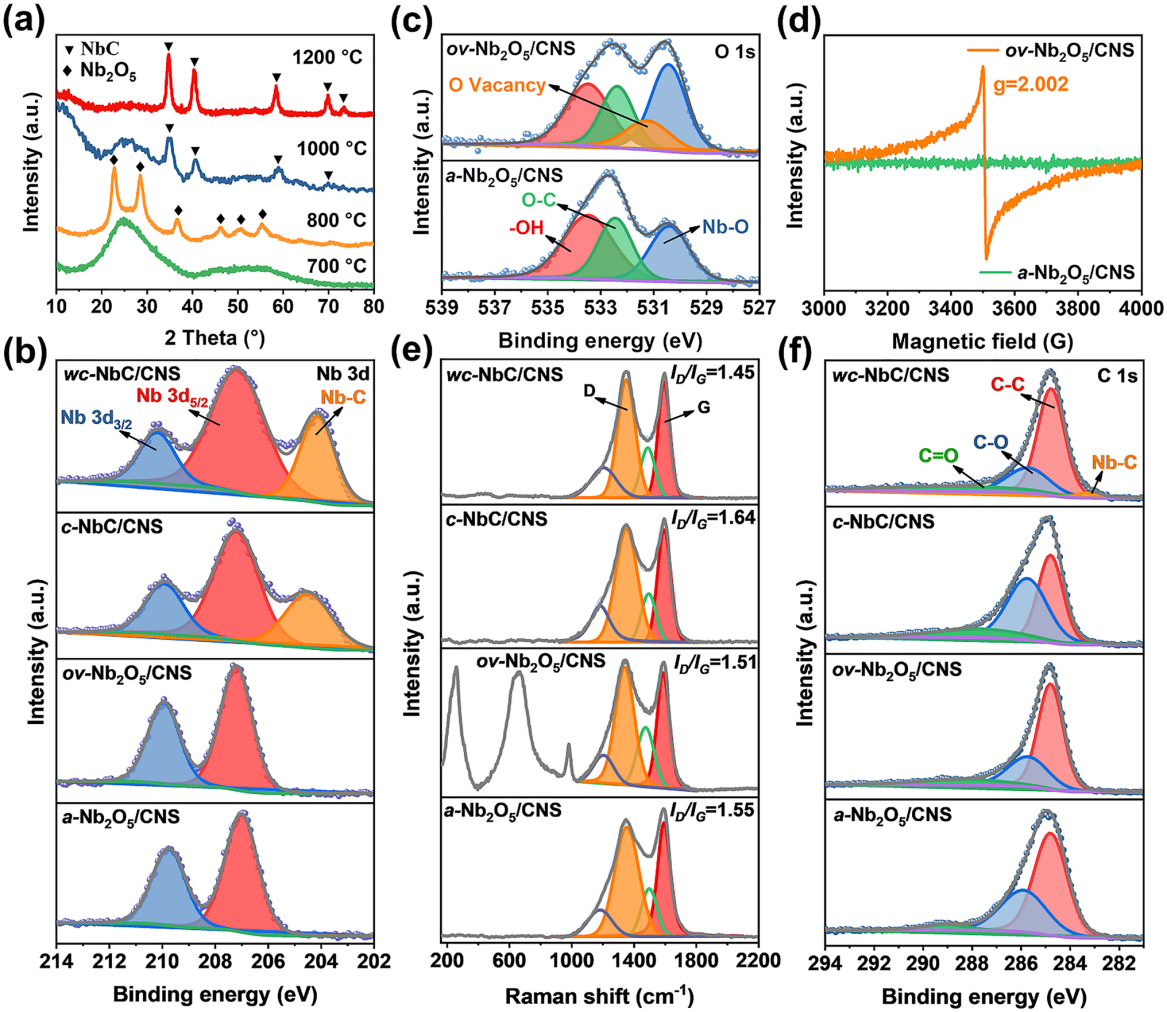
**a** X-ray diffraction (XRD) patterns and **b** high-resolution Nb 3*d* XPS spectra of all samples. **c** High-resolution O 1*s* XPS spectra and **d** EPR spectra of *a-*Nb_2_O_5_/CNS and *ov-*Nb_2_O_5_/CNS. **e** Raman spectra and **f** high-resolution C 1*s* XPS spectra images of all samples

The microcrystalline structures of all the samples can be disclosed by the X-ray diffraction (XRD) patterns (Fig. 3a). No characteristic peaks of Nb_2_O_5_ phases can be discerned in *a-*Nb_2_O_5_/CNS, confirming the existence of amorphous nanoparticles. The diffraction peaks in *ov-*Nb_2_O_5_/CNS are assigned to orthorhombic crystal Nb_2_O_5_ with a space group of Pbam (PDF, card No. 30–0873). For both *c-*NbC/CNS and *wc-*NbC/CNS, a group of typical sharp peaks corresponds to the cubic structure of the NbC nanocrystals (PDF, card No. 65–7964). The full width at half maximum (FWHM) of the characteristic NbC peaks decreases with increasing annealing temperature, confirming the growth of NbC nanocrystals. These results are consistent with the previous results obtained by HRTEM.

To reveal the elemental compositions and chemical states of the samples, X-ray photoelectron spectroscopy (XPS) analysis is performed. The survey spectrum confirms the coexistence of C, O, and Nb in the composites (Fig. S7). In the high-magnification Nb 3*d* XPS spectra of *a-*Nb_2_O_5_/CNS and *ov-*Nb_2_O_5_/CNS, two characteristic peaks of Nb_2_O_5_ at approximately 207.2 and 209.9 eV index to the doublet of Nb 3*d*_5/2_ and Nb 3*d*_3/2_, respectively (Fig. 3b) [40]. For *c-*NbC/CNS and *wc-*NbC/CNS, the formation of NbC can be determined by an extra peak at 204.7 eV in the Nb 3*d* spectra, which is attributed to the Nb-C bond. Additionally, after annealing at 800 °C, the presence of oxygen vacancies in Nb_2_O_5_ is confirmed by the sub-peak at approximately 531.2 eV in the O 1*s* spectra of *ov-*Nb_2_O_5_/CNS-800 (Fig. 3c) [41]. The oxygen vacancy defect sites are evidenced by electron paramagnetic resonance (EPR) analysis, as shown in Fig. 3d. A remarkable symmetric peak with a g-value of 2.002 ascribed to unpaired electrons can be observed for *ov-*Nb_2_O_5_/CNS [42], while no detectable EPR signal exists in Nb_2_O_5_/CNS-700. Moreover, the oxygen vacancy concentration of *ov-*Nb_2_O_5_/CNS quantitatively reaches 4.59 × 10^15^ spins mg^−1^.

The 2D lamellar carbon skeleton of all the samples can produce confinement to Nb-based nanoparticles to avoid aggregation and act as an in situ reductant to reduce Nb_2_O_5_ at a relatively high temperature. The graphitization degrees and structural defects of the composite 2D carbon skeletons are evaluated by Raman spectroscopy. As demonstrated in Fig. 3e, two distinct peaks at approximately 1340 and 1600 cm^−1^ are fitted into four sub-peaks to accurately identify the intensities of the D band and G band. The *I*_D_/*I*_G_ (area) values of *a-*Nb_2_O_5_/CNS, *ov-*Nb_2_O_5_/CNS, *c-*NbC/CNS, and *wc-*NbC/CNS are 1.55, 1.51, 1.64, and 1.45, respectively. The variation trend of *I*_D_/*I*_G_ is consistent with the intensity alteration of the C–O sub-peak in the high-resolution C 1*s* spectrum (Fig. 3f). These results imply that the presence of a large amount of amorphous Nb_2_O_5_ and the reduction in Nb_2_O_5_ can increase the structural defects and decrease the graphitization degree of the carbon skeleton.

### Electromagnetic Wave Absorption Properties

**Fig. 4:**
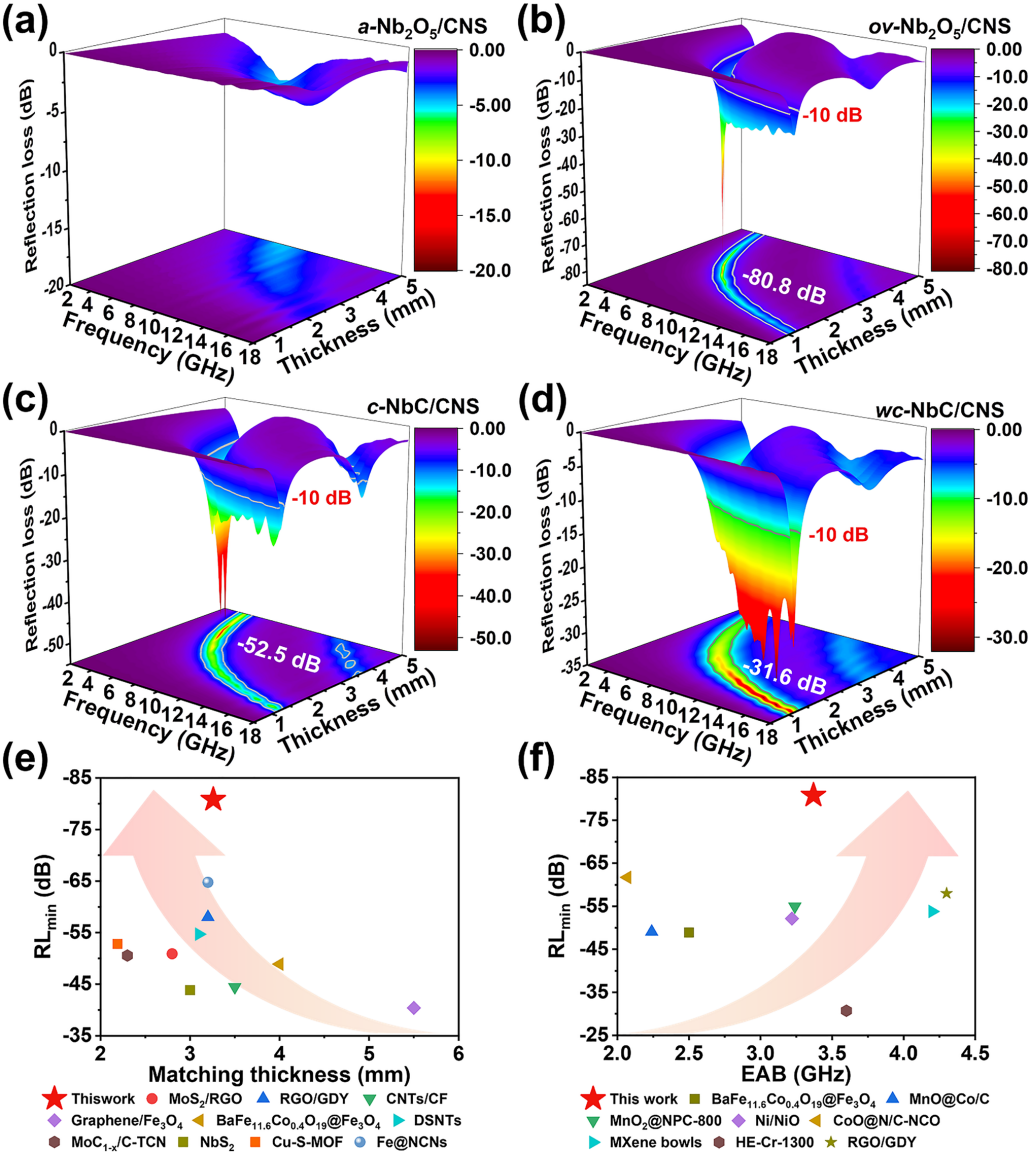
**a-d** Three-dimensional reflection loss diagrams of *a-*Nb_2_O_5_/CNS, *ov-*Nb_2_O_5_/CNS, *c-*NbC/CNS, and *wc-*NbC/CNS, respectively. Comparison of EMW absorption properties among *ov-*Nb_2_O_5_/CNS and other absorbers reported in the literature: **e** minimum reflection loss *versus* thickness and **f** minimum reflection loss *versus* effective absorption bandwidth

As-synthesized composites with monodispersed Nb-based nanoparticles and conductive 2D carbon skeletons have great potential in absorbing EMW. Therefore, the associated EMW absorption performance characteristics of *a-*Nb_2_O_5_/CNS, *ov-*Nb_2_O_5_/CNS, *c-*NbC/CNS, and *wc-*NbC/CNS composites are systematically evaluated (Fig. 4). The reflection loss (RL) coefficient based on transmission line theory is used to quantify the EMW absorption performance [43], which generally requires less than − 10 dB (> 90% wave absorption) to meet the demands of real-world applications. Notably, the *ov-*Nb_2_O_5_/CNS exhibits the most competitive absorption performance among the four samples, achieving an extremely low minimum RL (RL_min_) value of − 80.8 dB (> 99.999999% wave absorption) at 7.11 GHz, with a thickness of 2.76 mm (Fig. 4b). Moreover, *c-*NbC/CNS and *wc-*NbC/CNS deliver a relatively satisfactory absorbing performance of RL_min_ =  − 52.5 dB (5.08 GHz, 4.00 mm) and RL_min_ =  − 31.6 dB (16.32 GHz, 1.24 mm) (Fig. 4c–d). Unfortunately, *a-*Nb_2_O_5_/CNS demonstrates a disappointing EMW absorption performance of − 4.8 dB (Fig. 4a). The effective absorption bandwidth (EAB) values of *ov-*Nb_2_O_5_/CNS, *c-*NbC/CNS, and *wc-*NbC/CNS are 3.37 GHz (1.30 mm), 3.61 GHz (1.43 mm), and 4.41 GHz (1.30 mm), respectively (Fig. S8). Generally, *ov-*Nb_2_O_5_/CNS exhibits a high-attenuation *RL*_min_ of − 80.8 dB and a satisfactory EAB of 3.37 GHz, reflecting its extraordinary potential for EMW absorption.

Furthermore, a comparison of the EMW absorption performance between the *ov-*Nb_2_O_5_/CNS composite and other absorbers reported in the literature is conducted (Fig. 4e–f, Tables S2 and S3) [7, 9, 27, 44–56]. The as-prepared *ov-*Nb_2_O_5_/CNS composite exhibits ultrahigh EMW absorption performance with a thin matching thickness and a wide EAB. This phenomenon gives the composite a significant advantage over many other reported electromagnetic wave absorbers, making it an excellent choice for demanding EMW absorption applications.

### Electromagnetic Wave Absorption Mechanism

**Fig. 5:**
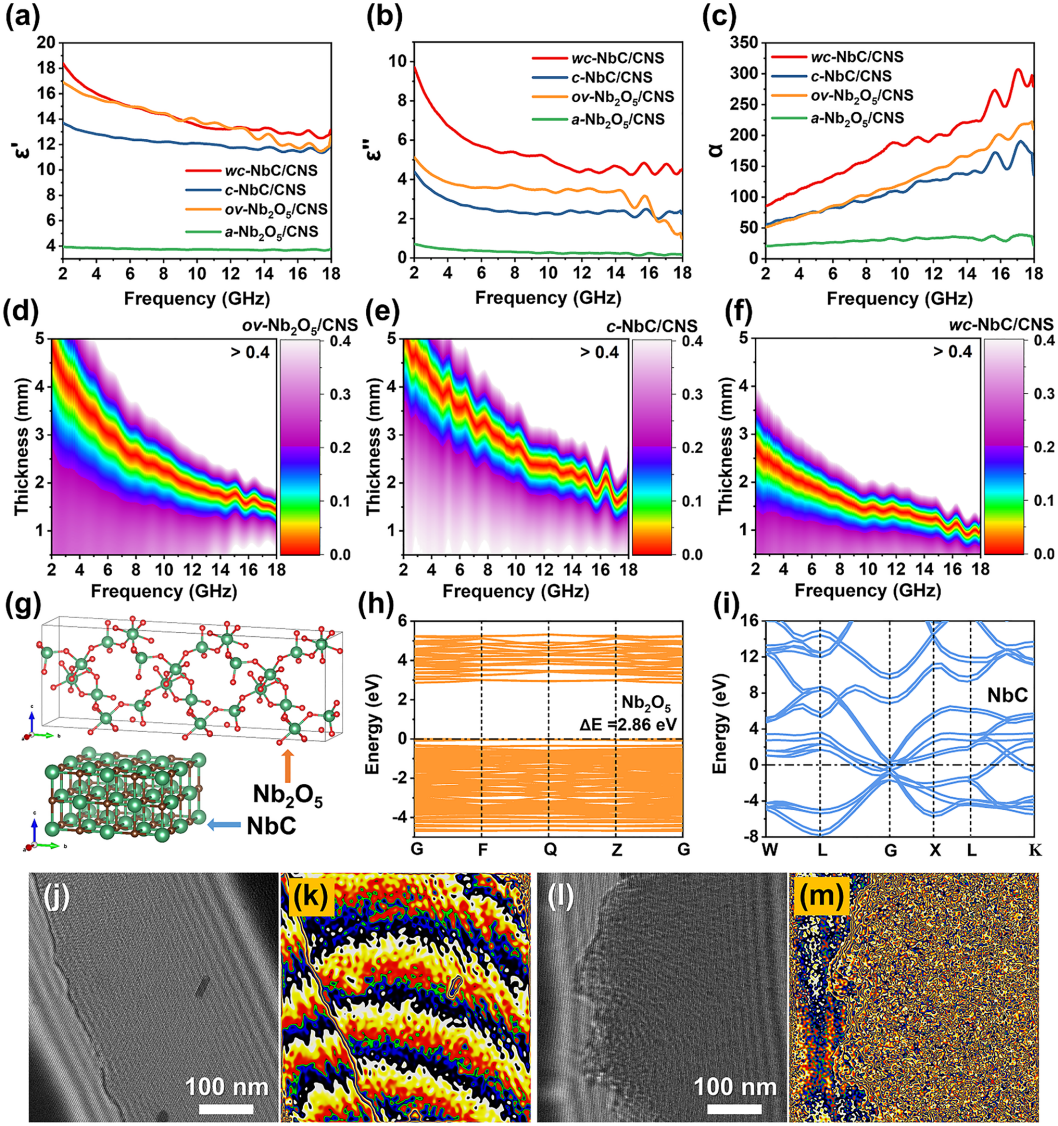
**a** Real part of permittivity, **b** imaginary part of permittivity, and **c** attenuation constant (*α*) of all samples. **d–f** 2D delta (|Δ|) value maps of *ov-*Nb_2_O_5_/CNS, *c-*NbC/CNS, and *wc-*NbC/CNS, respectively. **g** Structures of the Nb_2_O_5_ and NbC configurations. Energy band structures of **h** Nb_2_O_5_ and **i** NbC. **j** Off-axis electron holograms and **k** stray field flux lines of *ov-*Nb_2_O_5_/CNS. **l** Off-axis electron holograms and **m** stray field flux lines of *wc-*NbC/CNS

The EMW attenuation performance characteristics of MAMs are closely associated with the dielectric and magnetic loss parameters. As a pure dielectric system, the EMW absorbing performance characteristics of *a-*Nb_2_O_5_/CNS, *ov-*Nb_2_O_5_/CNS, *c-*NbC/CNS, and *wc-*NbC/CNS are primarily attributed to the value of the complex permittivity (*ε*_*r*_ = *ε*′ − *jε*″). The disappointing EMW absorption performance of *a-*Nb_2_O_5_/CNS can be reflected by the extremely low values of both *ε*′ and *ε*′′ (Fig. 5a-b). The *ε*′ for all the samples exhibits a generally decreasing trend as the frequency increases, which can be interpreted as the frequency dispersion behavior of the increased polarization hysteresis during the high-frequency electric field variation (Fig. 5a). Moreover, the multiple fluctuation peaks in *ε″* of *ov-*Nb_2_O_5_/CNS, *c-*NbC/CNS, and *wc-*NbC/CNS are assigned to the presence of polarization relaxation (Fig. 5b). Polarization relaxation can be induced by the electric dipoles in the Nb_2_O_5_ (or NbC) nanocrystals and interfacial polarizations at Nb_2_O_5_–carbon (or NbC–carbon) heterointerfaces. As shown in Fig. S9, the dielectric loss factor (tan*δ*_*ε*_) is applied to represent the EMW energy loss ability. Notably, *wc-*NbC/CNS has the highest tan*δ*_*ε*_, which may lead to impedance mismatching, thus weakening the microwave absorption performance.

The overall dissipation capabilities of these four samples to the incident microwave are further estimated by an attenuation constant (α) [4]. As illustrated in Fig. 5c, all the samples deliver *α* values with an overall upward trend of increasing frequency. The *ov-*Nb_2_O_5_/CNS composite delivers a larger α value than *a-*Nb_2_O_5_/CNS and *c-*NbC/CNS, implying its superior attenuation capacity. Conversely, the *wc-*NbC/CNS with the highest permittivity demonstrates an excessively large α value, potentially causing impedance mismatching. Then, a delta value (|Δ|) is calculated to evaluate the impedance matching [6], which represents the efficiency of EMW transmission into the absorber. The |Δ| values for *a-*Nb_2_O_5_/CNS are all above 0.4, demonstrating the extremely disappointing impedance matching ability (Fig. S10). Moreover, *wc-*NbC/CNS presents poor impedance matching, as indicated by the lowest integration area of |Δ| values smaller than 0.4 (Fig. 5f). Notably, *ov-*Nb_2_O_5_/CNS demonstrates the largest proper impedance matching area (rainbow-colored region, |Δ|< 0.2) (Fig. 5d), which suggests a high incident efficiency of EMW into the absorbers. Therefore, the superior impedance matching and enhanced attenuation capabilities of *ov-*Nb_2_O_5_/CNS are favorable for realizing high-attenuation EMW absorption.

The Nb-based nanoparticles in the composites operate as dielectric loss active components to improve the EMW absorption performance. To theoretically reveal the different dielectric behaviors of two different Nb-based species, first-principles calculations based on the energy band structure of Nb_2_O_5_ and NbC configurations are conducted (Fig. 5g). As shown in Fig. 5h, the electronic property of Nb_2_O_5_ is semiconductive, which can be identified by a forbidden gap with a small energy gap of 2.86 eV originating from the separated valence band and the conduction band. In contrast, the overlapping of the valence band and conduction band for NbC reveals its conductive nature (Fig. 5i). The poor impedance matching of *wc-*NbC/CNS may be attributed to the large amount of conductive NbC in the 2D carbon skeleton, which leads to excessively high permittivity parameters. Moreover, compared with NbC conductors, Nb_2_O_5_ semiconductors with reduced charge transfer ability slow the electrical neutralization of the dipole under an alternating electromagnetic field and hence reinforce dipole polarization relaxation. As a result, *ov-*Nb_2_O_5_/CNS with plenty of well-crystallized Nb_2_O_5_ semiconductors can achieve superior dielectric loss.

Nb_2_O_5_ and NbC with different dielectric behaviors can impact the electromagnetic responses of the composites. Off-axis electron holography is conducted to investigate the interaction of *ov-*Nb_2_O_5_/CNS and *wc-*NbC/CNS with the electromagnetic field (Fig. 5j–m). As shown in Fig. 5k, high-density stray field flux lines robustly penetrate from the inside of the *ov-*Nb_2_O_5_/CN nanosheet and radiate to the outside. This result confirms the significant interaction between the *ov-*Nb_2_O_5_/CNS composite and electromagnetic field. However, no obvious stray field flux lines are triggered to be released from *wc-*NbC/CNS (Fig. 5m). It can be speculated that the Nb_2_O_5_ nanoparticles produce plentiful dielectric polarization and activate a strong induced electromagnetic field to influence the EMW. Moreover, the divergence of stray field flux lines in *ov-*Nb_2_O_5_/CNS builds a multidimensional electromagnetic response network and effectively reinforces the attenuation of electromagnetic energy.

**Fig. 6:**
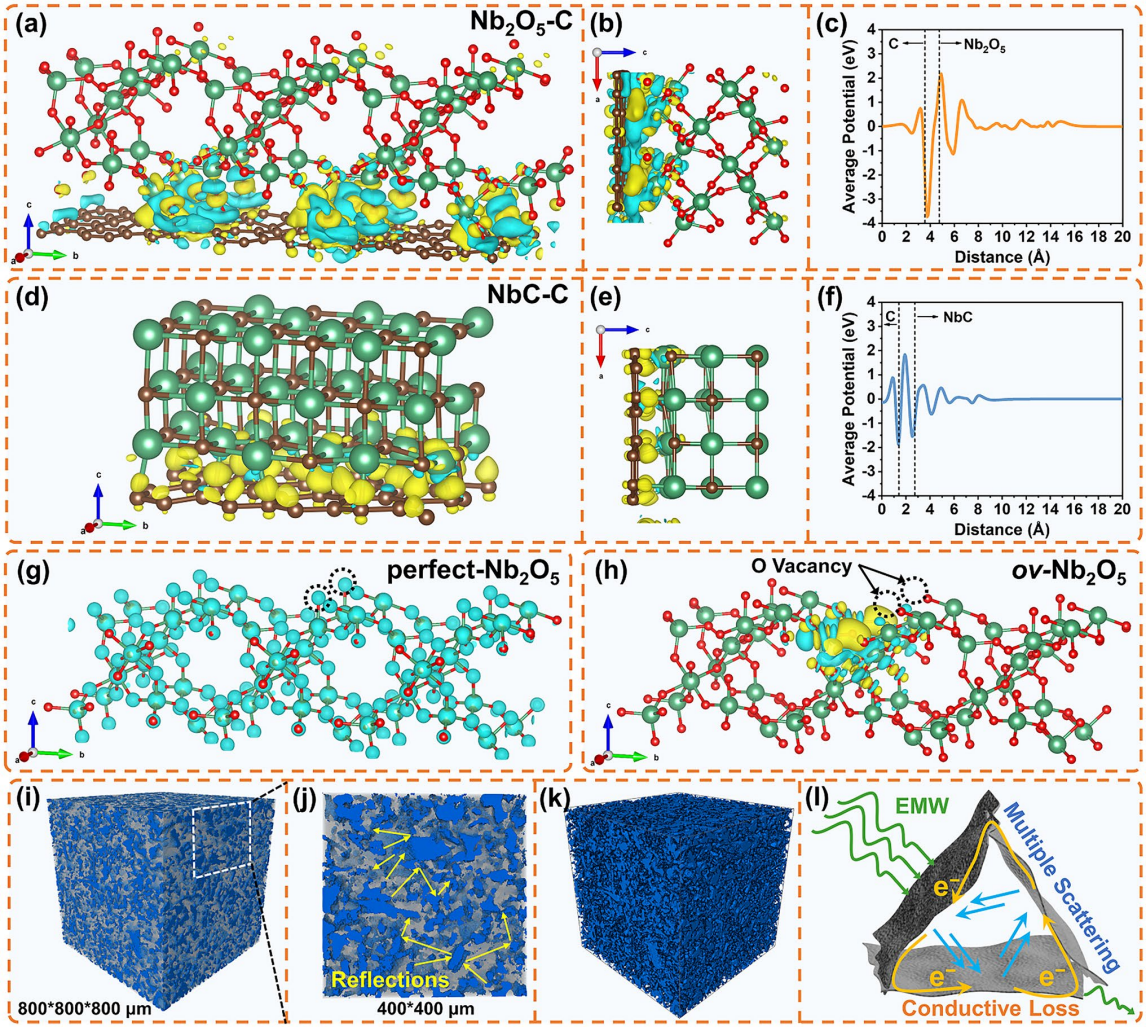
Charge density distribution of the **a–b** Nb_2_O_5_–carbon configuration and **d–e** NbC–carbon configuration. Planar average electrostatic potential of **c** Nb_2_O_5_–carbon and **f** NbC–carbon. **g** Charge density contour of perfect Nb_2_O_5_. **h** Charge density distribution of *ov-*Nb_2_O_5_ (yellow and blue regions represent aggregation and dispersion of electrons). **i-k** Micro-CT images of the *ov-*Nb_2_O_5_/CNS-paraffin absorber by 3D reconstruction (dark blue and gray regions refer to the *ov-*Nb_2_O_5_/CNS and paraffin wax phase, respectively). **l** Schematic illustrations of the multiple scattering and conductive loss mechanism of the 2D lamellar *ov-*Nb_2_O_5_/CNS. (Color figure online)

Moreover, the incorporation of Nb_2_O_5_ and NbC nanoparticles into the carbon nanosheets generates multiple heterointerfaces. Interfacial charge aggregation occurs due to the disrupted original electric balance at these heterointerfaces, which typically leads to interfacial polarization. Notably, Nb_2_O_5_ and NbC with different electronic properties can affect the charge aggregation at Nb_2_O_5_–carbon and NbC–carbon interfaces, respectively. Therefore, the charge density distributions of the Nb_2_O_5_–carbon and NbC–carbon configurations are investigated to theoretically reveal the interfacial polarization induced by Nb-based nanoparticles (Figs. 6a–f and S11). As depicted in Fig. 6a, b, d, e, charges are unevenly distributed at these heterointerfaces, where irregular yellow and blue regions correspond to the aggregation and dispersion of electrons, respectively. It is apparent that the electrons generally delocalize from the carbon and flow into Nb_2_O_5_ at the Nb_2_O_5_–carbon heterointerface, while the delocalized electrons flow from the carbon and NbC to the intermediate region at the NbC–carbon interface. These results are supported by the planar average potential of the Nb_2_O_5_–carbon and NbC–carbon interfaces (Fig. 6c, f). Moreover, the Nb_2_O_5_–carbon heterointerface demonstrates a pronounced charge separation effect, which consequently enhances the interfacial polarization capability. The *ov-*Nb_2_O_5_/CNS with abundant well-crystallized Nb_2_O_5_ nanoparticles possesses numerous Nb_2_O_5_–carbon heterointerfaces. Consequently, intensified electromagnetic energy dissipation through strengthened interfacial polarization loss can be achieved by *ov-*Nb_2_O_5_/CNS.

Complementary to excellent interfacial polarization loss, the presence of abundant oxygen vacancy defects in Nb_2_O_5_ nanoparticles can contribute to the excellent electric dipole polarization in *ov-*Nb_2_O_5_/CNS. The charge distributions of perfect Nb_2_O_5_ and oxygen vacancy Nb_2_O_5_ configurations are estimated by first-principles calculations to better understand the intensification of electric dipole polarization for *ov-*Nb_2_O_5_/CNS. Figure S12 presents the configurations of perfect Nb_2_O_5_ and oxygen vacancy Nb_2_O_5_ (*ov-*Nb_2_O_5_). The charge distribution in perfect Nb_2_O_5_ is relatively uniformly distributed (Fig. 6g). However, with the presence of two oxygen vacancies, electrons delocalize at the vacancy sites and flow into nearby oxygen atoms, resulting in charge separation (Fig. 6h). Subsequently, electric dipoles are generated in *ov-*Nb_2_O_5_, which can induce the formation of electronic dipole polarization oscillation in an external electromagnetic field. Therefore, Nb_2_O_5_ nanoparticles with abundant oxygen vacancies can act as electronic dipole polarization oscillation units, thereby efficiently enhancing the dielectric loss capability of *ov-*Nb_2_O_5_/CNS.

To gain a comprehensive understanding of the dielectric loss mechanisms, Cole–Cole plots based on Debye theory are further investigated. As depicted in Fig. S13, the rich polarization relaxation processes for the *ov-*Nb_2_O_5_/CNS are suggested by a more perfect Cole–Cole semicircle with a large diameter. In contrast, the highly distorted Cole–Cole trajectories observed for other composites suggest a weakened Debye dipolar relaxation.

Furthermore, the 2D lamellar morphology of *ov-*Nb_2_O_5_/CNS is beneficial for establishing abundant nanosheet–paraffin interfaces, which can strengthen the multiple reflection and scattering dissipation characteristics. To enable 3D reconstruction of the phase diagram, the *ov-*Nb_2_O_5_/CNS-paraffin absorber is scanned using a microcomputer tomography (micro-CT) device, as shown in Fig. 6i–j. Image slicing techniques are employed to acquire the nondestructive *ov-*Nb_2_O_5_/CNS phase (blue phase) and paraffin phase (transparent gray phase). The *ov-*Nb_2_O_5_/CNS nanosheets stack in the absorber and form numerous voids, and these voids are filled with paraffin wax. This unique structure can produce multiple macro-heterointerfaces to enhance interfacial polarization loss and prolong the reflection and scattering path of the EMW to enhance attenuation. Additionally, upon removing the paraffin phase, an interconnected 3D conductive *ov-*Nb_2_O_5_/CNS network is observed (Fig. 6k), which can realize intensive conductivity loss. Generally, these results suggest that the *ov-*Nb_2_O_5_/CNS in the absorber can efficiently strengthen the attenuation of EMW by intensifying interfacial polarization loss, multiple reflections, scattering dissipation, and conductivity loss.

### Possible Application Prospects

**Fig. 7:**
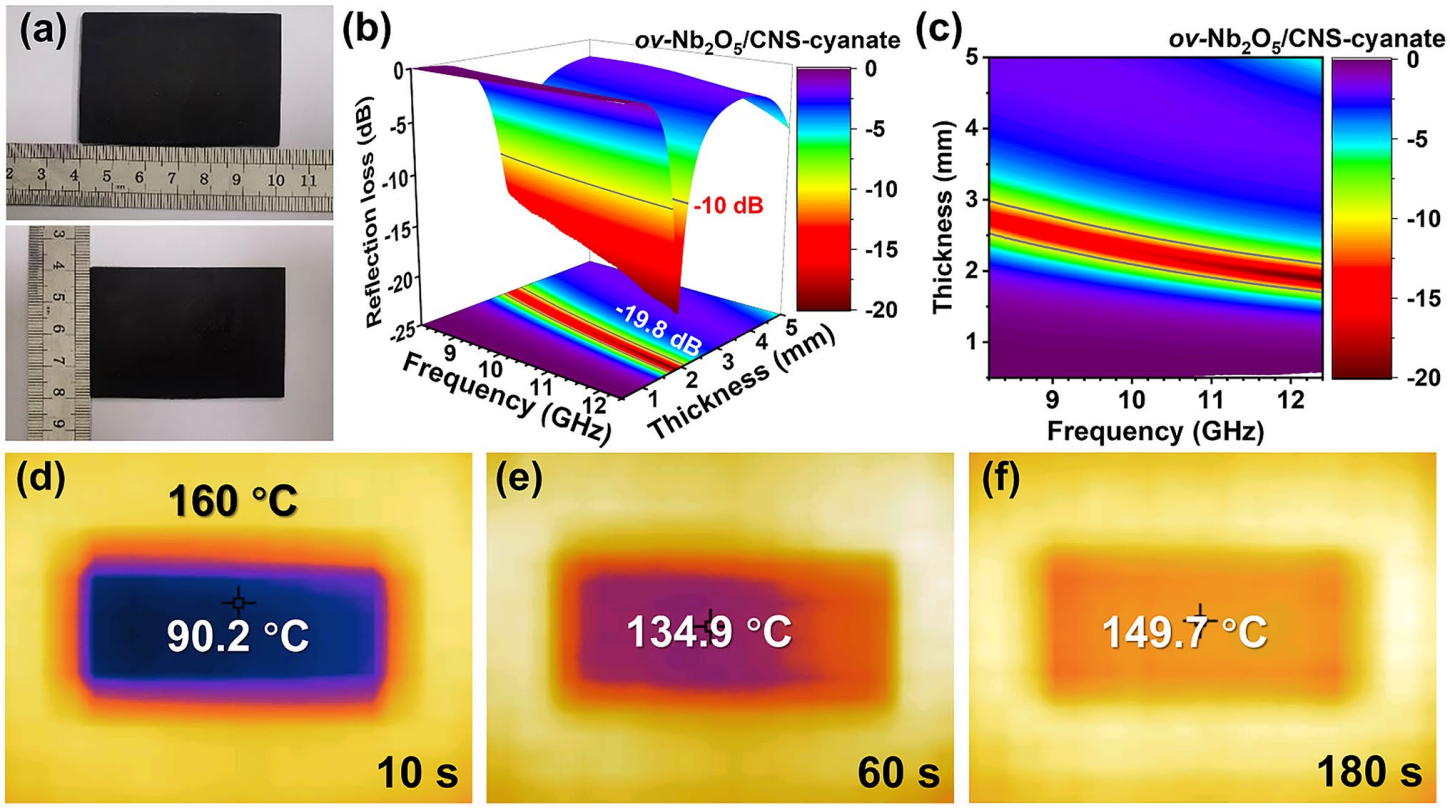
**a** Digital photographs of the *ov-*Nb_2_O_5_/CNS-cyanate plate. **b** Three-dimensional reflection loss diagrams and **c** reflection loss contour maps of the *ov-*Nb_2_O_5_/CNS-cyanate plate. **d–f** Thermal infrared images of the *ov-*Nb_2_O_5_/CNS-cyanate plate on a heating platform (160 °C)

In practical applications, EMW absorbing materials are mainly used as coatings or plates. To verify the application potential of *ov-*Nb_2_O_5_/CNS, the composite is mixed with EMW-transmitting cyanate ester to form a mixed resin. The functional resin can successfully cure into a rectangular plate (80 mm × 40 mm × 2 mm), as shown in Fig. 7a. Moreover, the *ov-*Nb_2_O_5_/CNS-cyanate plate demonstrates excellent machinability, allowing it to be precisely cut to a size of 22.9 mm × 10.2 mm × 2 mm to investigate the microwave absorption performance using a waveguide method (Fig. S14). As shown in Figs. 7b–c and S15, the *ov-*Nb_2_O_5_/CNS-cyanate plate achieves a satisfying RL_min_ value of − 19.8 dB at 12.38 GHz with a thickness of 1.88 mm, and the EAB reaches 2.41 GHz with a thickness of 2.10 mm. Since the absorption mechanism of EMW absorbers is dominated by transforming electromagnetic energy into thermal energy, the heat dissipation capacity is an imperative factor in practical applications. When the *ov-*Nb_2_O_5_/CNS-cyanate plate is placed on a heating platform (160 °C), the temperature of the plate increases rapidly to 90.2 °C (56.4% of 160 °C) within 10 s and reaches a stable temperature of approximately 150 °C (93.8% of 160 °C) after 180 s (Figs. 7d–f and S16). Overall, *ov-*Nb_2_O_5_/CNS-800 demonstrates excellent application potential by curing into an excellent microwave-absorbing, machinable, and heat-dissipating *ov-*Nb_2_O_5_/CNS-cyanate plate.

## Conclusions

In conclusion, we demonstrate the successful synthesis of ultrafine (~ 10 nm) Nb_2_O_5_ nano-semiconductors with rich oxygen vacancies in carbon nanosheets (*ov-*Nb_2_O_5_/CNS) for achieving high-attenuation electromagnetic wave absorption. Semiconductive Nb_2_O_5_ nanoparticles endow *ov-*Nb_2_O_5_/CNS with more strengthened charge polarization at the Nb_2_O_5_–carbon hetero-interface than NbC conductors, which facilitates interfacial polarization loss. Additionally, Nb_2_O_5_ nanocrystals with abundant oxygen vacancies reinforce electric dipole polarization inside the semiconductor. The 2D lamellar morphology of *ov-*Nb_2_O_5_/CNS strengthens the multiple reflection and scattering dissipation characteristics in the absorbers. Therefore, when integrated with outstanding polarization relaxation, intensified electromagnetic response, and excellent impedance matching, *ov-*Nb_2_O_5_/CNS achieves a superior EMW absorption capability with an unparalleled *RL*_min_ of − 80.8 dB (> 99.999999% wave absorption) at 7.11 GHz (2.76 mm); additionally, it exhibits a wide effective absorption bandwidth of 3.37 GHz at 1.30 mm. Moreover, the composite shows excellent application potential by curing into a microwave-absorbing, machinable, and heat-dissipating *ov-*Nb_2_O_5_/CNS-cyanate plate. The *ov-*Nb_2_O_5_/CNS-cyanate plate achieves a satisfactory RL_min_ value of − 19.8 dB at 12.38 GHz with a thickness of 1.88 mm, and the EAB reaches 2.41 GHz with a thickness of 2.10 mm. Our findings provide novel insights into the design and development of dielectric semiconductor-based carbon composites for electromagnetic wave absorption and related applications.

### Supplementary Information

Below is the link to the electronic supplementary material.

Supplementary file1 (PDF 1515 KB)
