# Supplementary material for: Ultrafine Vacancy-Rich Nb2O5 Semiconductors Confined in Carbon Nanosheets Boost Dielectric Polarization for High-Attenuation Microwave Absorption
Source: Nanomicro Lett. 2023 Jul 14;15:183. doi: 10.1007/s40820-023-01151-0 (PMC10349032; doi:10.1007/s40820-023-01151-0)
Supplement: Supplementary file 1 — Supplementary file1 (PDF 1515 KB) [file 40820_2023_1151_MOESM1_ESM.pdf]

Supporting Information for

Ultrafine Vacancy-Rich Nb_2O_5 Semiconductors Confined in Carbon Nanosheets Boost Dielectric Polarization for High-Attenuation Microwave Absorption

Zhe Su[1], Shan Yi[1], Wanyu Zhang[1], Xiaxi Xu[1], Yayun Zhang[1], Shenghu Zhou[1], Bo Niu[1, *], and Donghui Long[1, 2, *]

[1] Shanghai Key Laboratory of Multiphase Materials Chemical Engineering, East China University of Science and Technology, School of Chemical Engineering, Shanghai 200237, P. R. China

[2] Key Laboratory for Specially Functional Materials and Related Technology of the Ministry of Education, East China University of Science and Technology, Shanghai 200237, P. R. China

*Corresponding authors. E-mail: longdh@mail.ecust.edu.cn (Donghui Long), niubo@ecust.edu.cn (Bo Niu)

ORCID: Donghui Long, orcid.org/0000-0002-3179-4822

Bo Niu, orcid.org/0000-0003-4126-6246

S1 Supplementary Calculation Section

According to the transmission line theory, the values of RL are calculated via the following equations [1]:

$$R_L(\text{dB}) = 20\log\left|\frac{Z_{in}-Z_0}{Z_{in}+Z_0}\right| \quad \text{(S1)}$$

$$Z_{in} = Z_0\sqrt{\frac{\mu_r}{\varepsilon_r}}\tanh\left[j\frac{2\pi}{c}\sqrt{\mu_r\varepsilon_r}fd\right] \quad \text{(S2)}$$

where Z_0 is the impedance of free space, Z_{in} is the normalized input impedance of the absorber, ε_r ($\varepsilon_r=\varepsilon' - j\varepsilon''$) is the relative complex permittivity of the absorber, μ_r ($\mu_r=\mu' - j\mu''$) is the relative complex permeability, f represents the electromagnetic wave frequency, c is the velocity of the EM wave in free space, and d expresses the thickness of the absorber, respectively.

According to Debye dipolar relaxation (Cole-Cole model), the permittivity can be described as [S2]:

$$\varepsilon_r = \varepsilon' - j\varepsilon'' = \varepsilon_\infty + \frac{\varepsilon_S-\varepsilon_\infty}{1+j2\pi f\tau} \quad \text{(S3)}$$

where τ, ε_s, ε_∞, and f stands for the polarization relaxation time, static permittivity constant, relative dielectric permittivity at the infinite frequency, and frequency, respectively. And the ε' and ε'' can be deduced as follows:

$$\varepsilon' = \varepsilon_\infty + \frac{\varepsilon_S-\varepsilon_\infty}{1+(2\pi f\tau)^2} \quad \text{(S4)}$$

$$\varepsilon'' = \varepsilon_\infty + \frac{2\pi f\tau(\varepsilon_S-\varepsilon_\infty)}{1+(2\pi f\tau)^2} \quad \text{(S5)}$$

$$\left(\varepsilon' - \frac{\varepsilon_S+\varepsilon_\infty}{2}\right)^2 + (\varepsilon'')^2 = \left(\frac{\varepsilon_S-\varepsilon_\infty}{2}\right)^2 \quad \text{(S6)}$$

The attenuation constant α can be evaluated through the following equation [S3]:

$$\alpha = \frac{\sqrt{2}\pi f}{c} \times \sqrt{(\mu''\varepsilon'' - \mu'\varepsilon') + \sqrt{(\mu''\varepsilon'' - \mu'\varepsilon')^2 + (\mu'\varepsilon'' + \mu''\varepsilon')^2}} \quad \text{(S7)}$$

Delta values (|Δ|) can be deduced by the following equations [S2],

$$|\Delta| = |\sinh^2(Kfd) - M| \quad \text{(S8)}$$

where K and M can be determined by the relative complex permittivity and permeability via following equation,

$$\mathrm{K} = \frac{4\pi\sqrt{\mu'\varepsilon'}\cdot\sin(\frac{\delta_e+\delta_m}{2})}{c\cdot\cos\delta_e\cdot\cos\delta_m} \quad \text{(S9)}$$

$$\mathrm{M} = \frac{4\mu'\varepsilon'\cos\delta_e\cos\delta_m}{(\mu'\cos\delta_e - \varepsilon'\cos\delta_m)^2 + \left[\tan\left(\frac{\delta_m}{2} - \frac{\delta_e}{2}\right)\right]^2(\mu'\cos\delta_e + \varepsilon'\cos\delta_m)^2} \quad \text{(S10)}$$

S2 Supplementary Figures and Tables

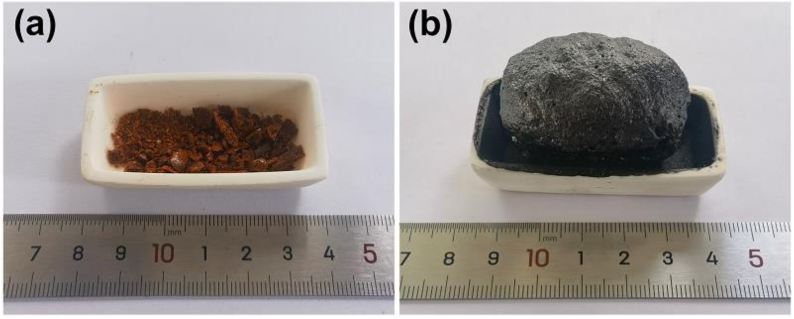

Fig. S1 The digital photographs of **a** Nb^{5+}-gluconate precursor and **b** foamed *ov*-Nb_2O_5/CNS

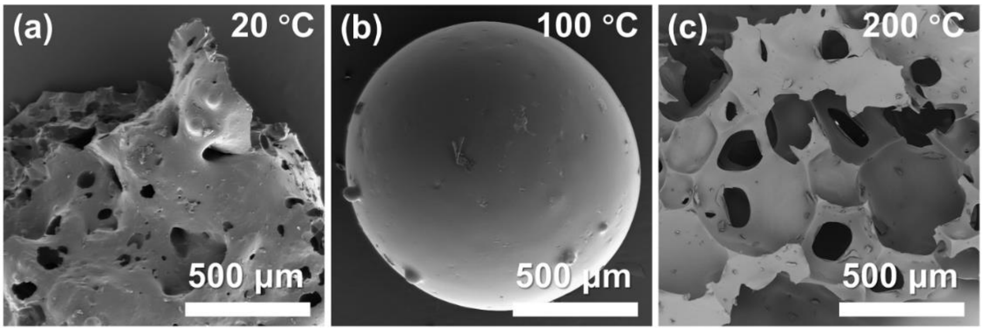

Fig. S2. The scanning electron microscope (SEM) images of **a** Nb^{5+}-gluconate precursor at 20 °C, **b** melted Nb^{5+}-gluconate precursor at 100 °C, and **c** foamed intermediates with closed-cell structures at 200 °C

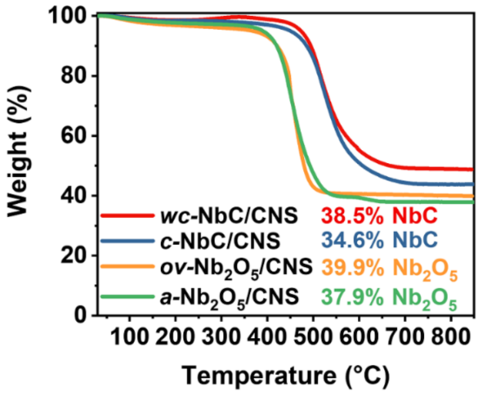

Fig. S3 Thermogravimetric analysis (TGA) curves of *a*-Nb_2O_5/CNS, *ov*-Nb_2O_5/CNS, *c*-NbC/CNS, and *wc*-NbC/CNS composites under an air atmosphere

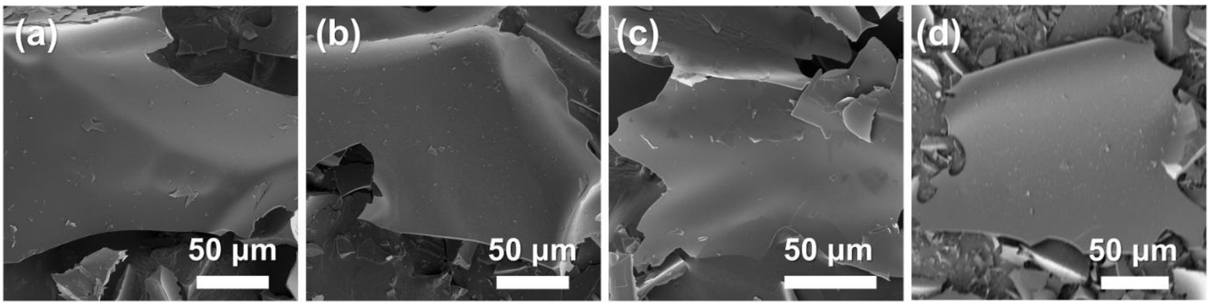

Fig. S4 SEM images of **a** *a*-Nb_2O_5/CNS, **b** *ov*-Nb_2O_5/CNS, **c** *c*-NbC/CNS, and **d** *wc*-NbC/CNS composites

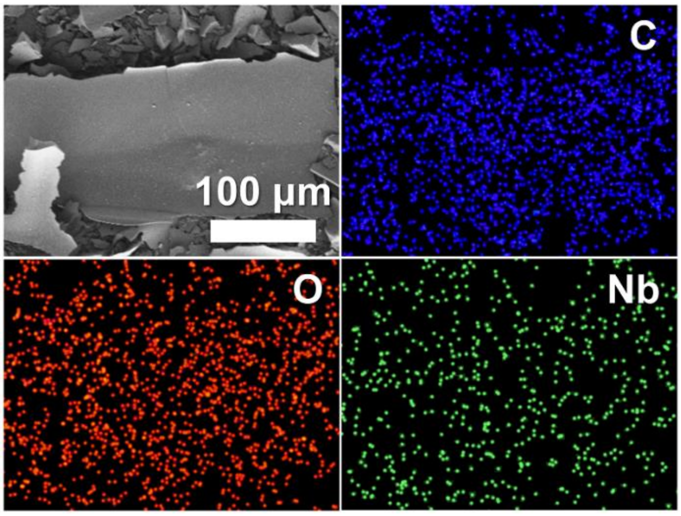

Fig. S5 X-ray energy-dispersive spectroscopy (EDS) images of *ov*-Nb_2O_5/CNS

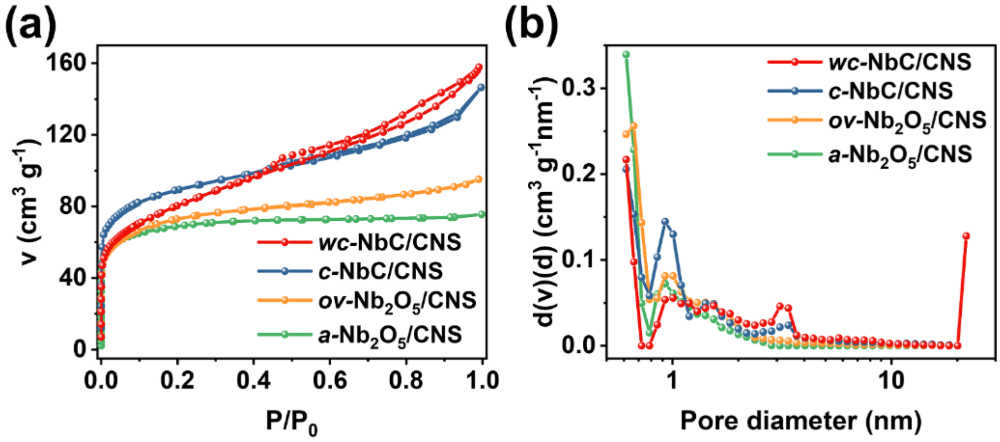

Fig. S6 a N_2 adsorption/desorption isotherms and **b** corresponding QSDFT pore-size distribution curves of *a*-Nb_2O_5/CNS, *ov*-Nb_2O_5/CNS, *c*-NbC/CNS, and *wc*-NbC/CNS composites

The reduction of Nb_2O_5 by the carbon skeleton and the subsequent growth of NbC crystals results in the generation of a greater number of mesopores. This phenomenon is evidenced by the N_2 adsorption/desorption isotherms of c-NbC/CNS and *wc*-NbC/CNS composites, which display an enlarged hysteresis loop at higher relative pressures (P/P_0 of 0.4-0.99).

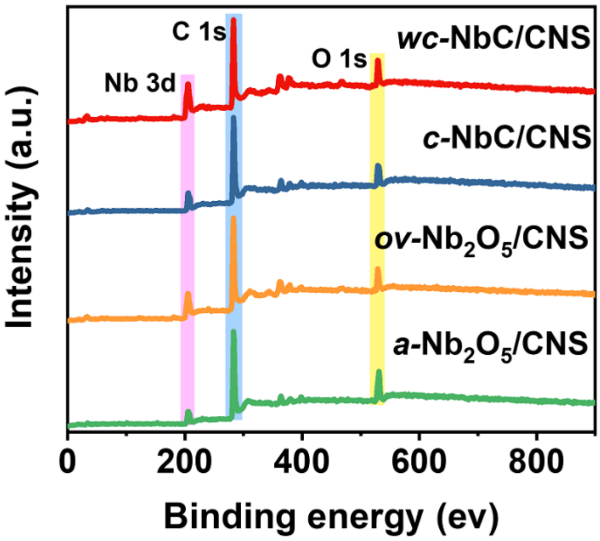

Fig. S7 XPS survey spectrum of *a*-Nb_2O_5/CNS, *ov*-Nb_2O_5/CNS, *c*-NbC/CNS, and *wc*-NbC/CNS composites

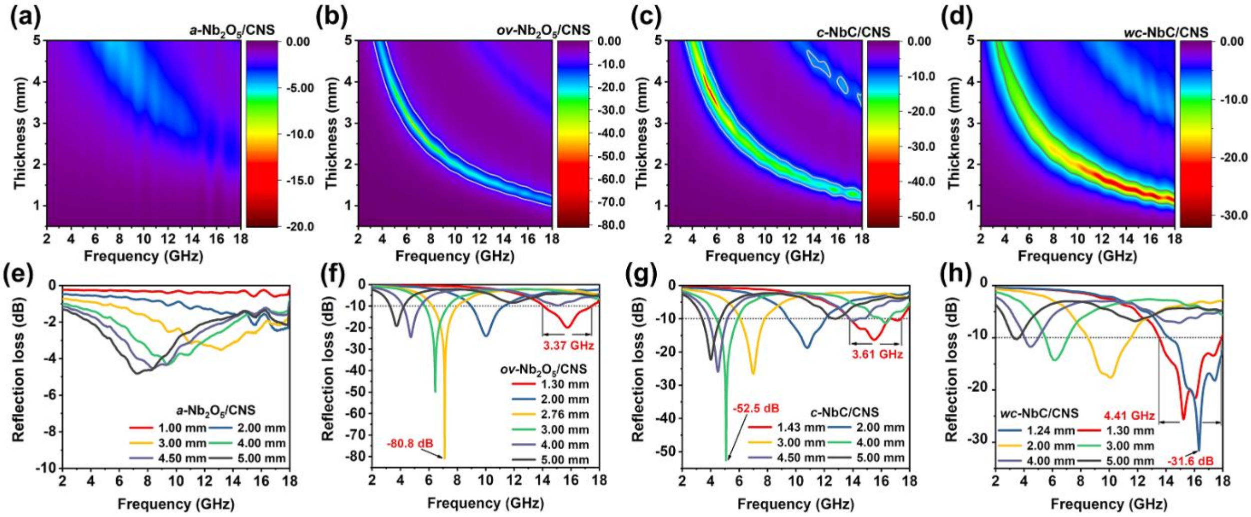

Fig. S8 Reflection loss contour maps and corresponding curves of **a,e** *a*-Nb_2O_5/CNS, **b,f** *ov*-Nb_2O_5/CNS, **c,g** *c*-NbC/CNS, and **d,h** *wc*-NbC/CNS composites

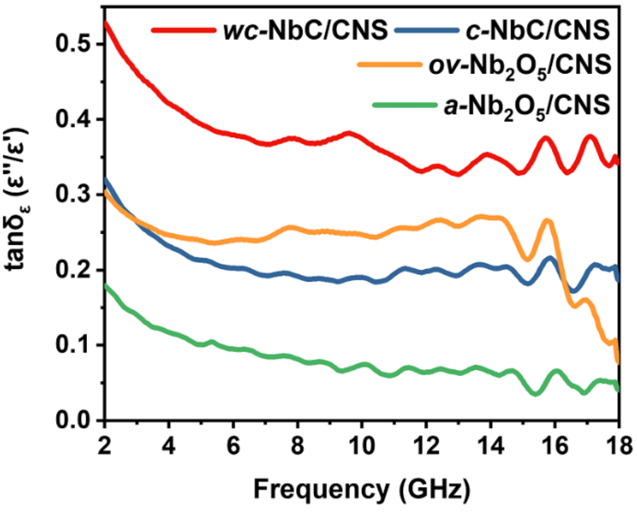

Fig. S9 Dielectric loss factor ($\tan\delta_\varepsilon$) of *a*-Nb_2O_5/CNS, *ov*-Nb_2O_5/CNS, *c*-NbC/CNS, and *wc*-NbC/CNS

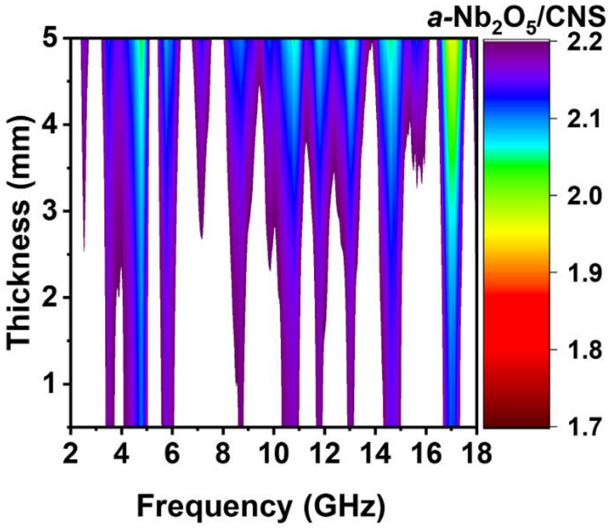

Fig. S10 2D delta (|Δ|) value maps of *a*-Nb_2O_5/CNS

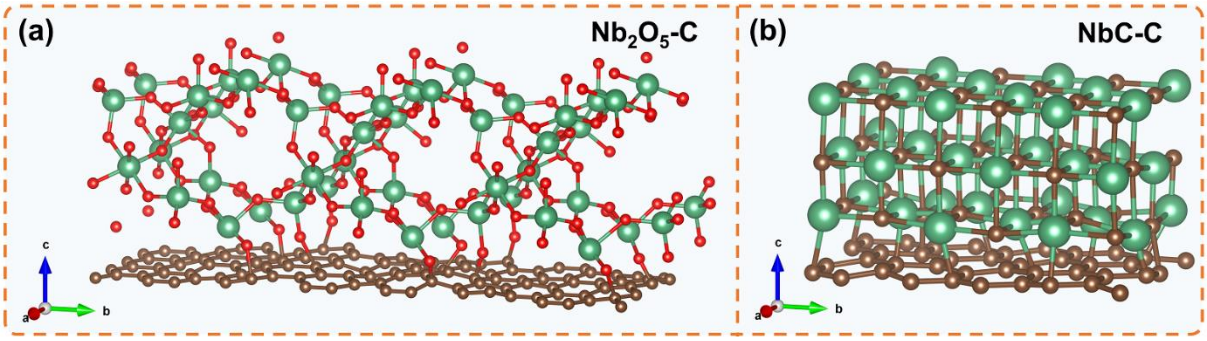

Fig. S11 Structures of **a** Nb_2O_5–carbon and **b** NbC–carbon configurations

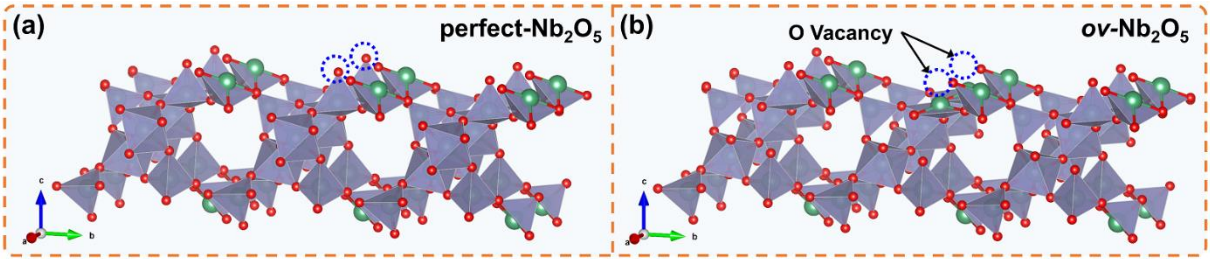

Fig. S12 Structures of **a** perfect Nb_2O_5 and **b** oxygen-vacancy Nb_2O_5 configurations

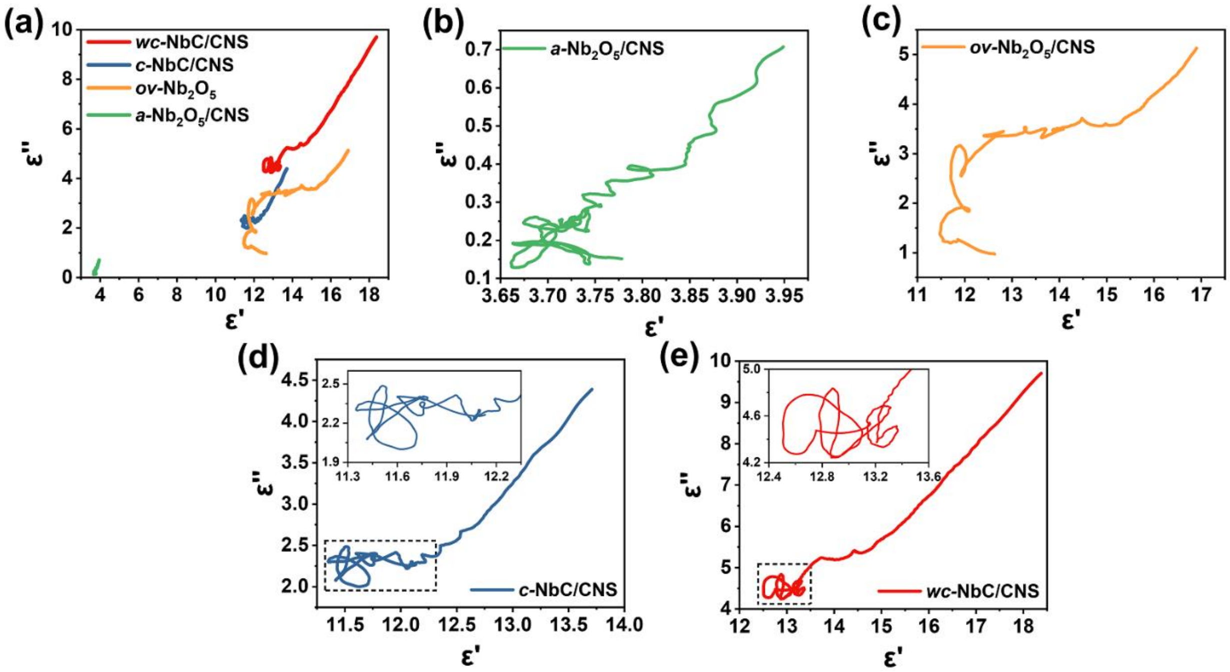

Fig. S13 **a** Comprehensive and **b-e** individual Cole-Cole plots of *a*-Nb_2O_5/CNS, *ov*-Nb_2O_5/CNS, *c*-NbC/CNS, and *wc*-NbC/CNS

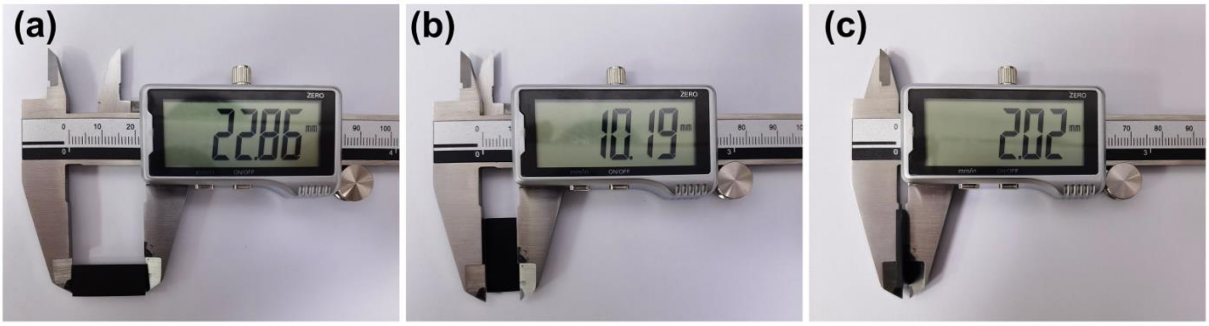

Fig. S14 The digital photographs of *ov*-Nb_2O_5/CNS-cyanate plate with a size of 22.9 mm×10.2 mm×2 mm

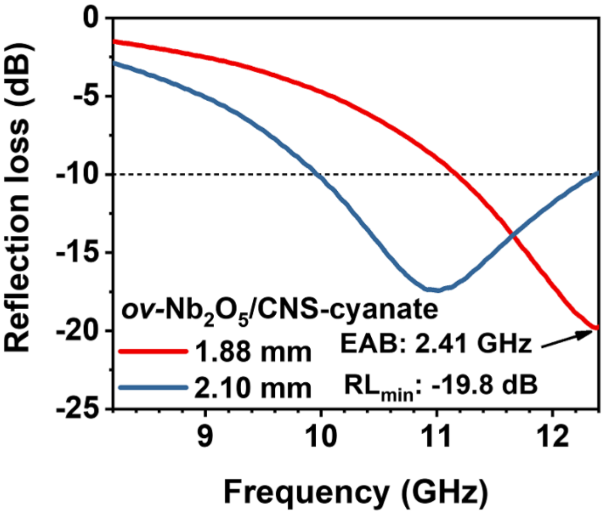

Fig. S15 The detailed reflection losses curve of *ov*-Nb_2O_5/CNS-cyanate plate

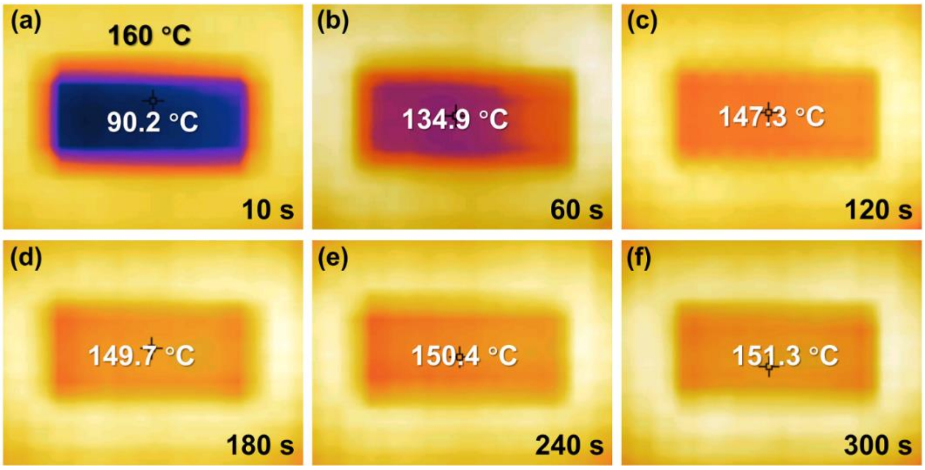

Fig. S16 Thermal infrared images of *ov*-Nb_2O_5/CNS-cyanate plate on a heating platform (160 °C)

Table S1 Detailed pore parameters of *a*-Nb_2O_5/CNS, *ov*-Nb_2O_5/CNS, *c*-NbC/CNS, and *wc*-NbC/CNS

**Sample**	**S_{BET} (m^2 g^{-1})**	**S_{Micro} (m^2 g^{-1})**	**V_{Total} (cm^3 g^{-1})**	**V_{Micro} (cm^3 g^{-1})**
a-Nb_2O_5/CNS	224.2	201.9	0.117	0.098
ov-Nb_2O_5/CNS	262.3	216.4	0.147	0.095
c-NbC/CNS	314.9	215.5	0.227	0.096
wc-NbC/CNS	278.3	112.0	0.244	0.054

Table S2 Comparison of minimum reflection loss *versus* thickness among *ov*-Nb_2O_5/CNS and other absorbers reported in the literature

Sample	Minimum reflection loss (dB)	Thickness (mm)	References
ov-Nb_2O_5/CNS	−80.8	2.76	This work
MoS_2/RGO	−50.9	2.3	[S4]
RGO/GDY	−58	2.7	[S5]
CNTs/CF	−44.46	3	[S6]
Graphene/Fe_3O_4	−40.4	5	[S7]
$BaFe_{11.6}Co_{0.4}O_{19}$@$Fe_3O_4$	−48.9	3.5	[S8]
DSNTs	−54.7	2.6	[S9]
MoC_{1-x}/C-TCN	−50.55	1.8	[S10]
NbS_2	−43.85	2.5	[S11]
Cu-S-MOF	−52.8	1.69	[S12]
Fe@NCNs	−64.75	2.7	[S13]

Table S3 Comparison of minimum reflection loss *versus* effective absorption bandwidth among *ov*-Nb_2O_5/CNS and other absorbers reported in the literature

Sample	Minimum reflection loss (dB)	Effective absorption bandwidth (GHz)	References
ov-Nb_2O_5/CNS	−80.8	3.37	This work
$BaFe_{11.6}Co_{0.4}O_{19}$@$Fe_3O_4$	−48.9	2.5	[S8]
MnO@Co/C	−49.06	2.24	[S14]
MnO_2@NPC-800	−54.96	3.24	[S15]
Ni/NiO	−52.15	3.22	[S16]
CoO@N/C-NCO	−61.73	2.07	[S17]
MXene bowls	−53.8	4.2	[S18]
HE-Cr-1300	−30.7	3.6	[S19]
RGO/GDY	−58	4.3	[S5]

Supplementary References

[S1] J. Cheng, H. Zhang, M. Ning, H. Raza, D. Zhang, G. Zheng, Q. Zheng, R. Che. Emerging materials and designs for low-and multi-band electromagnetic wave absorbers: the search for dielectric and magnetic synergy? Adv. Funct. Mater. **32**(23), 2200123 (2022). https://doi.org/10.1002/adfm.202200123

[S2] X. Zeng, X. Cheng, R. Yu, G. D. Stucky. Electromagnetic microwave absorption theory and recent achievements in microwave absorbers. Carbon **168**, 606-623 (2020). https://doi.org/10.1016/j.carbon.2020.07.028

[S3] M. S. Cao, X. X. Wang, M. Zhang, J. C. Shu, W. Q. Cao, H. J. Yang, X. Y. Fang, J. Yuan. Electromagnetic response and energy conversion for functions and devices in low-dimensional materials. Adv. Funct. Mater. **29**(25), 1807398 (2019). https://doi.org/10.1002/adfm.201807398

[S4] Y. Wang, D. Chen, X. Yin, P. Xu, F. Wu, M. He. Hybrid of MoS_2 and reduced graphene oxide: a lightweight and broadband electromagnetic wave absorber. ACS Appl. Mater. Interfaces **7**(47), 26226-26234 (2015). https://doi.org/10.1021/acsami.5b08410

[S5] M. Ling, F. Wu, P. Liu, Q. Zhang, B. Zhang. Fabrication of graphdiyne/graphene composite microsphere with wrinkled surface via ultrasonic spray compounding and its microwave absorption properties. Small **19**(7), 2205925 (2023). https://doi.org/10.1002/smll.202205925

[S6] Y. Zhan, L. Xia, H. Yang, N. Zhou, G. Ma, T. Zhang, X. Huang, L. Xiong, C. Qin, W. Guangwu. Tunable electromagnetic wave absorbing properties of carbon nanotubes/carbon fiber composites synthesized directly and rapidly via an innovative induction heating technique. Carbon. **175**, 101-111 (2021). https://doi.org/10.1016/j.carbon.2020.12.080

[S7] T. Wang, Z. Liu, M. Lu, B. Wen, Q. Ouyang, Y. Chen, C. Zhu, P. Gao, C. Li, M. Cao. Graphene-Fe_3O_4 nanohybrids: synthesis and excellent electromagnetic absorption properties. J. Appl. Phys. **113**(2), 024314 (2013). https://doi.org/10.1063/1.4774243

[S8] M. Yuan, B. Zhao, C. Yang, K. Pei, L. Wang, R. Zhang, W. You, X. Liu, X. Zhang, R. Che. Remarkable magnetic exchange coupling via constructing bi-magnetic interface for broadband lower-frequency microwave absorption. Adv. Funct. Mater. **32**(33), 2203161 (2022). https://doi.org/10.1002/adfm.202203161

[S9] P. Liu, Y. Wang, G. Zhang, Y. Huang, R. Zhang, X. Liu, X. Zhang, R. Che. Hierarchical engineering of double-shelled nanotubes toward hetero-interfaces induced polarization and microscale magnetic interaction. Adv. Funct. Mater. **32**(33), 2202588 (2022). https://doi.org/10.1002/adfm.202202588

[S10] T. Zhao, Z. Jia, Y. Zhang, G. Wu. Multiphase molybdenum carbide doped carbon hollow sphere engineering: the superiority of unique double-shell structure in microwave absorption. Small **19**(6), 2206323 (2023). https://doi.org/10.1002/smll.202206323

[S11] H. Zhang, J. Cheng, H. Wang, Z. Huang, Q. Zheng, G. Zheng, D. Zhang, R. Che, M. Cao. Initiating VB-group laminated NbS_2 electromagnetic wave absorber toward superior absorption bandwidth as large as 6.48 GHz through phase engineering modulation. Adv. Funct. Mater. **32**(6), 2108194 (2022). https://doi.org/10.1002/adfm.202108194

[S12] P. Miao, N. Qu, W. Chen, T. Wang, W. Zhao, J. Kong. A two-dimensional semiconductive Cu-S metal-organic framework for broadband microwave absorption. Chem. Eng. J. **454**, 140445 (2023). https://doi.org/10.1016/j.cej.2022.140445

[S13] T. Gao, R. Zhao, Y. Li, Z. Zhu, C. Hu, L. Ji, J. Zhang, X. Zhang. Sub-nanometer Fe clusters confined in carbon nanocages for boosting dielectric polarization and broadband electromagnetic wave absorption. Adv. Funct. Mater. **32**(31), 2204370 (2022). https://doi.org/10.1002/adfm.202204370

[S14] M. Liu, R. Tian, H. Chen, S. Li, F. Huang, K. Peng, H. Zhang. One-dimensional chain-like MnO@Co/C composites for high-efficient electromagnetic wave absorbent. J. Magn. Magn. Mater. **499**, 166289 (2020). https://doi.org/10.1016/j.jmmm.2019.166289

[S15] H. Yang, Z. Shen, H. Peng, Z. Xiong, C. Liu, Y. Xie. 1D-3D mixed-dimensional MnO_2@nanoporous carbon composites derived from Mn-metal organic framework with full-band ultra-strong microwave absorption response. Chem. Eng. J. **417**, 128087 (2021). https://doi.org/10.1016/j.cej.2020.128087

[S16] Y. Shu, T. Zhao, X. Li, L. Yang, S. Cao. Enhanced electromagnetic wave absorption properties integrating diverse loss mechanism of 3D porous Ni/NiO microspheres. J. Alloy. Compd. **897**, 163227 (2022). https://doi.org/10.1016/j.jallcom.2021.163227

[S17] W. Wang, H. Zhang, Y. Zhao, J. Wang, H. Zhao, P. Li, J. Yun, Z. Deng, Z. Zhang, J. Tian. A novel MOF-drived self-decomposition strategy for CoO@N/C-Co/Ni-$NiCo_2O_4$ multi-heterostructure composite as high-performance electromagnetic wave absorbing materials. Chem. Eng. J. **426**, 131667 (2021). https://doi.org/10.1016/j.cej.2021.131667

[S18] Q. Du, Q. Men, R. Li, Y. Cheng, B. Zhao, R. Che. Electrostatic adsorption enables layer stacking thickness-dependent hollow $Ti_3C_2T_x$ MXene bowls for superior electromagnetic wave absorption. Small **18**(47), 2203609 (2022). https://doi.org/10.1002/smll.202203609

[S19] B. Zhao, Y. Du, Z. Yan, L. Rao, G. Chen, M. Yuan, L. Yang, J. Zhang, R. Che. Structural defects in phase-regulated high-entropy oxides toward superior microwave absorption properties. Adv. Funct. Mater. **33**(1), 2209924 (2023). https://doi.org/10.1002/adfm.202209924
